# A multivariate correlated poisson generalized inverse gaussian regression model for dependent count data: Estimation and testing procedures

**DOI:** 10.1016/j.mex.2025.103772

**Published:** 2025-12-17

**Authors:** Yusrianti Hanike, Purhadi, Achmad Choiruddin

**Affiliations:** a Department of Statistics, Universitas Islam Negeri Abdul Muthalib Sangaji Ambon, Sirimau, Kota Ambon 97128, Indonesia; b Department of Statistics, Institut Teknologi Sepuluh Nopember, Sukolilo, Surabaya 60111, Indonesia

**Keywords:** Berndt-hall-hall-hausman (bhhh), Maximum likelihood ratio test (mlrt), Multivariate correlated poisson generalized inverse gaussian (mcpgig), Maternal mortality, Neonatal mortality

## Abstract

Regression modeling for multivariate count data often struggles with assumption of overdispersion and correlation among response variables. To address these issues, this study proposes a new model called Multivariate Correlated Poisson Generalized Inverse Gaussian Regression (MCPGIGR), which integrates random effects through common shock variables and allows for flexible mean structures via a log-link function. This research develops a Maximum Likelihood Estimation (MLE) and Maximum Likelihood Ratio Tests (MLRT) to evaluate both simultaneous and partial significance of predictors. We conduct simulation studies to assess the consistency and performance of the proposed estimators. Furthermore, in an application to maternal and neonatal mortality across 38 districts/cities in East Java (Indonesia), MCPGIGR substantially improves model fit relative to a Multivariate Poisson Regression (MPR) baseline (AICc decreases from 2378.63 to 1924.60 for γ=−1/2). The proposed framework provides a practical and flexible tool for analyzing correlated, overdispersed multivariate counts in public health and related domains. The highlights of this research are:

• The MCPGIGR model introduces a correlated multivariate count regression framework with exposure adjustment.

• It provides robust parameter estimation and hypothesis testing via MLE and MLRT.

• MCPGIGR demonstrates improved model fit and practical interpretability in public health applications.

## Graphical abstract

**Figure:**
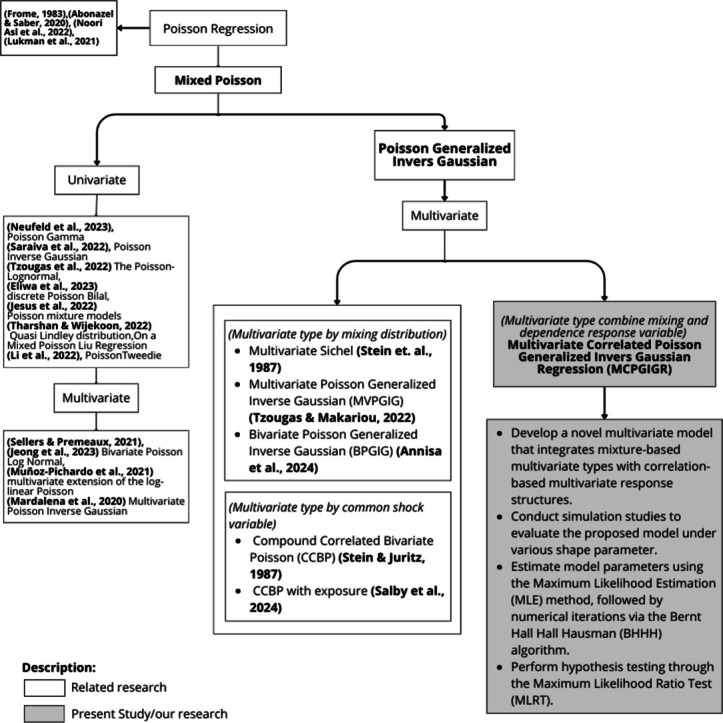

## Specifications table

**Table:** 

**Subject area**	Mathematics and Statistics
**More specific subject area**	Statistics; Multivariate Analysis; Count Data; Maximum Likelihood
**Name of your method**	Multivariate Correlated Poisson Generalized Inverse Gaussian Regression (MCPGIGR)
**Name and reference of original method**	Stein, G. Z., & Juritz, J. M. (1987). Bivariate compound Poisson distributions. Communications in Statistics - Theory and Methods, 16(12), 3591–3607. https://doi.org/10.1080/03610928708829593
**Resource availability**	Maternal and neonatal mortality data used in this study were obtained from the Health Profile of East Java Province 2023, published by the East Java Provincial Health Office. The dataset includes annual health indicators for all districts/municipalities in East Java. The full report can be accessed at the official website of the East Java Provincial Health Office:
	https://dinkes.jatimprov.go.id/index.php?r=site/file_list&id_file=10&id_berita=8
	Direct download link to the document:
	https://dinkes.jatimprov.go.id/userfile/dokumen/PROFIL%20KESEHATAN%20PROVINSI%20JAWA%20TIMUR%20TAHUN%202023.pdf

## Background

Poisson regression is a workhorse for count data and has been applied in many disciplines, such as traffic accidents [1,2], maternal and infant mortality [[3], [4], [5]], and the spread of infectious diseases [6,7]. In practice, however, two features frequently violate the standard Poisson assumptions: overdispersion and dependence across outcomes. Many modern applications also involve multiple, potentially correlated counts, which calls for multivariate modeling rather than separate univariate fits.

Several strategies have been developed to induce dependence in multivariate Poisson models. A classical construction uses a common-shock component: in the bivariate case, $\left(Y_{1},Y_{2}\right)=\left(Z_{1}+Z_{0},Z_{2}+Z_{0}\right)$, where $Z_{1},Z_{2},Z_{0}$, and are independent Poisson random variables [8,9]. In this perspective, the rate of the latent Poisson random variable $Z_{0}$, referred to as the common shock variable, dictates the strength of correlation between the two variables. Broader reviews of multivariate Poisson formulations are provided by [10]. The application of copulas for modeling multivariate discrete data has also been explored extensively by researchers such as [11,12]. Copulas offer flexibility but present complexities in discrete contexts. Mixed models for counts are popular [[13], [14], [15]], yet capturing cross-margin dependence typically requires careful distributional choices and may remain limited in flexibility. Therefore, it is crucial to consider the development of more flexible distributions, such as the mixed Poisson, which can provide a more adaptive and representative solution for phenomena that exhibit unobserved heterogeneity [16,17].

In this study, we build on the mixed-Poisson framework with a Poisson Generalized Inverse Gaussian (PGIG) mixing distribution and extend it to a multivariate correlated regression model, termed Multivariate Correlated Poisson Generalized Inverse Gaussian Regression (MCPGIGR). Dependence is induced through the common-shock mechanism, while GIG mixing on the Poisson rate accommodates overdispersion and yields a flexible dependence structure. The mean specification retains the interpretable log link with exposure, which is standard in applied count regression For half-integer [18,19].

Parameters are estimated by maximum likelihood (MLE) using the BHHH algorithm (Berndt–Hall–Hall–Hausman), which relies on per-observation score vectors [20,21]. We conduct maximum likelihood ratio test (MLRT) for simultaneous parameter testing and Wald Z test for partial testing. To aid accessibility, we present derivations in the main text and compile the detailed steps linking the pmf to the log-likelihood and score, including the Bessel identities in Appendix A.

Contributions of this paper are to (i) formulate MCPGIGR model, a correlated multivariate count regression with exposures and likelihood-based testing; (ii) derive estimation procedure with efficient BHHH estimation; (iii) demonstrate performance through simulations and an application to maternal and neonatal mortality in East Java, where MCPGIGR improves fit over a multivariate Poisson baseline.

This article is organized as follows: Section 2 introduces the distribution, the MCPGIGR regression specification, and the parameter estimation and testing procedures; Section 3 presents the simulations and the East Java application and concludes with brief implementation notes; Appendix A provides detailed derivations.

## Methodological details

Parameter estimation and hypothesis testing for MCPGIGR begin with formulation of the multivariate Poisson and GIG, which mixes a multivariate Poisson distribution with a GIG random effect to induce dependence and handle overdispersion. Building on this distribution, we specify the MCPGIG regression with a log link and involve exposure variable. Parameters are estimated by MLE using the BHHH algorithm, and statistical tests are derived using MLRT for joint-parameter test and Wald Z-test for individual test. Detailed derivations are presented in Appendix A.

### Multivariate poisson distribution

The Poisson distribution is a standard model for count data and may be viewed as a limiting form of the Binomial distribution. Typical settings involve rare events observed over a fixed interval (time/area) and independent trials [22]. Let Y be a Poisson random variable with parameter λ and exposure q. Its probability mass function (pmf) [22]:

(1) $$P\left(Y=y\right)=\frac{e^{-\lambda (q)}\lambda {\left(q\right)}^{y}}{y!},y=0,1,2,.......;\lambda \left(q\right)>0$$

with E(Y)=Var(Y). In regression applications, λ represents the expected number of events per unit exposure; q scales the population at risk or observation time [14].

A multivariate Poisson model specifies a joint distribution for two or more Poisson responses that may be correlated. A classical construction is the (m+1) variate reduction (common-shock) approach [[23], [24], [25]]. Let $Z_{1},Z_{2},\ldots ,Z_{j};j=1,2,...,m$ be mutually independent random variables, each following a Poisson distribution with respective parameters $\lambda_{1}\left(q_{1}\right),\lambda_{2}\left(q_{2}\right),...,\lambda_{j}\left(q_{j}\right);j=1,2,...,m$, where $q_{j}$ is defined as exposure variable.

Define new random variables $Y_{1},Y_{2},...,Y_{j}$ as follows:

(2) $$Y_{1}=Z_{1},Y_{2}=Z_{1}+Z_{2},\ldots ,Y_{j}=Z_{1}+Z_{j};j=2,\ldots ,m$$

the joint pmf of the Multivariate Poisson distribution (see **Appendix A.1**), $\lambda (q)=[\lambda_{1}\left(q_{1}\right)\;\lambda_{2}\left(q_{2}\right)\;...\;\lambda_{j}\left(q_{j}\right)]$ and $y=[\begin{array}{cccc} y_{1} & y_{2} & ... & y_{j} \end{array}]$ can be expressed as:

(3) $$P\left(y|\lambda (q)\right)=\prod_{j=2}^{m}\frac{{\left(\lambda_{j}\left(q_{j}\right)\right)}^{y_{j}-y_{1}}}{\left(y_{j}-y_{1}\right)!}\exp\left(-\left(\lambda_{1}\left(q_{1}\right)+\sum_{j=2}^{m}\lambda_{j}\left(q_{j}\right)\right)\right)\frac{{\left(\lambda_{1}\left(q_{1}\right)\right)}^{y_{1}}}{y_{1}!};y_{1},y_{2},...,y_{j}=0,1,2,...$$

with $y_{j}\geq y_{1};j=2,3,...,m$. The mean and variance of each random variable $Y_{j}$, are respectively, given by: $E\left(Y_{j}\right)=Var\left(Y_{j}\right)=\lambda_{j}q_{j}+\lambda_{1}q_{1}$. These expressions showed that the correlation among components arises entirely from the shared component $Z_{1}$.

### Multivariate correlated poisson generalized inverse gaussian distribution

The MCPGIG is model is a mixed Multivariate Poisson which includes correlated response variables. The response variables, $Y_{1},Y_{2},...,Y_{j};j=1,2,...,m$, $Y_{j}∼Poisson\left(\lambda_{j}\left(q_{j}\right)\right),j=1,2,...,m,$ assuming their means and variances are identical. The conditional variance can be greater than the conditional mean resulting from positive contagion and unobserved heterogeneity. An error term $\varepsilon_{j}$ is added to $\lambda_{j}$,

(4) $$e^{\left(X^{T}\beta_{j}+\varepsilon_{j}\right)}=\lambda_{j}\left(q_{j}\right)e^{\varepsilon_{j}}=\lambda_{j}\left(q_{j}\right)\nu$$

thus, $\lambda_{1}\left(q_{1}\right)\nu ,\lambda_{2}\left(q_{2}\right)\nu ,...,\lambda_{j}\left(q_{j}\right)\nu ;j=1,2,...,m$ represent the means for each response variable now, where $Y_{j}∼\text{Mixed}\text{Poisson}\left(\lambda_{j}\left(q_{j}\right)\nu \right)$ [14,26]. The characteristics of the mixed Poisson distribution rely on the specific distribution of the random variable ν. In this study, ν follows a GIG distribution. Therefore, $Y_{1},Y_{2},...,Y_{j};j=1,2,...,m$ follows a mixed Poisson distribution based on (4), (3), probability density function (pdf) of GIG is provided in [27]. In probability theory and statistics, the g(ν;τ,ξ,γ) distribution is a three-parameter continuous probability distribution with a pdf [18] as follows:

(5) $$g\left(\nu ;\tau ,\xi ,\gamma \right)=\frac{\xi^{-\gamma }}{2K_{\gamma }\left(\sqrt{\tau^{2}+\xi^{2}}-\xi \right)}\nu^{\gamma -1}\exp\left(-\left(\frac{\sqrt{\tau^{2}+\xi^{2}}-\xi }{2}\right)\left(\frac{\nu }{\xi }+\frac{\xi }{\nu }\right)\right),\nu >0$$

where −∞<γ<∞,τ>0,ξ>0, $K_{\gamma }\left(\sqrt{\tau^{2}+\xi^{2}}-\xi \right)$ is the third kind modified Bessel function o γ [28]. Here, the parameter γ regulates the tail behavior and controls the Poisson–GIG mixing variance, while ξ reflects the scale component that interacts with the latent mixing variable. The parameter τ represents the dispersion level of the GIG random effect, influencing the variability propagated into the mixed Poisson rates. These parameters jointly determine the degree of overdispersion and the flexibility of the MCPGIG distribution. The result of MCPGIG distribution (see **Appendix A.2**) based on the integral table by [29] as mentioned in [30], can be stated on:

(6) $$P\left(y|\lambda \left(q\right),\tau ,\xi ,\gamma \right)=\frac{\xi^{-\gamma }{\left(\lambda_{1}\left(q_{1}\right)\right)}^{y_{1}}}{K_{\gamma }\left(\varpi \right)y_{1}!}\prod_{j=2}^{m}\frac{{\left(\lambda_{j}\left(q_{j}\right)\right)}^{y_{j}-y_{1}}}{\left(y_{j}-y_{1}\right)!}{\left(\frac{\varpi \xi^{2}}{\psi }\right)}^{\frac{y_{j}+\gamma }{2}}K_{{}_{y_{j}+\gamma }}\left(\sqrt{\psi \varpi }\right);y_{1},y_{2},...,y_{j}=0,1,2,...$$

with $\varpi =\sqrt{\tau^{2}+\xi^{2}}-\xi ;\psi =\left(2\xi (\lambda_{1}\left(q_{1}\right)+\sum_{j=2}^{m}\lambda_{j}\left(q_{j}\right))+\varpi \right)$ where −∞<γ<∞;τ>0; $\lambda_{j}\left(q_{j}\right)>0;y_{j}>y_{1};j=2,3,...,m$. Eq. (6) is obtained by integrating the MP pmf in Eq. (3) with respect to the GIG mixing density in Eq. (5). Specifically, the MCPGIG pmf follows from the mixing identity (see **Appendix A.2** in equation (A.2.2)) where P(y|λ(q)ν) is the Poisson component with mixed rate $\lambda_{j}\left(q_{j}\right)\nu$ and g(ν;τ,ξ,γ) is the GIG density. The mean and variance of $Y_{j}$ $E\left(Y_{j}\right)=\xi \left(\lambda_{j}\left(q_{j}\right)+\lambda_{1}\left(q_{1}\right)\right)R_{\gamma }\left(\varpi \right)$,$Var\left(Y_{j}\right)=\left(\lambda_{j}\left(q_{j}\right)+\lambda_{1}\left(q_{1}\right)\right)\xi^{2}\frac{K_{\gamma +2}\left(\varpi \right)}{K_{\gamma }\left(\varpi \right)}+E\left(Y_{j}\right)\left(1-E(Y_{j})\right)$.

The MCPGIGR, if response variable $\left(Y_{i1},Y_{i2},...,Y_{ij}\right)∼MCPGIG\left(\lambda_{j}\left(q_{ij}\right)\right),$ where i=1,2,...,n and j=1,2,...,m then the MCPGIGR model can be stated as follows:

(7) $$E\left(Y_{ij}\right)=q_{ij}e^{x_{i}^{T}\beta_{j}}$$

with $q_{ij}$ is an exposure variable, defined as the weight of the observation for the *i* th and *j*-th units. Let $x_{i}^{T}=\left[1\;x_{i1}\;x_{i2}\ldots \;x_{ik}\right]$, k=1,2,...,p(i=1,2,...,n) be the vector of predictor variables with a dimension of (p+1) for the *i* th observation, and $\beta_{j}^{T}=\left[\begin{array}{ccc} \begin{array}{cc} \begin{array}{cc} \beta_{j0} & \beta_{j1} \end{array} & \beta_{j2} \end{array} & ... & \beta_{jk} \end{array}\right]$, j=1,2,...,m;k=1,2,...,p be a (p+1)×1 vector of regression coefficients associated with the *j*-th response variable.

The MCPGIGR model combines induced correlation (via a common-shock component) with GIG mixing on the Poisson rate, thereby accommodating both overdispersion and cross response dependence. The mean structure retains the interpretable log link, while additional variability is captured through the GIG parameters.

### Parameter estimation of mcpgigr model

The MLE method is employed for parameter estimation. This estimation method aims to determine the parameter values that yield the greatest probability for generating the observed data. This estimation method can be used when the pmf is known. The requirement for the MLE estimation method is that the samples are independent. The likelihood function of the MCPGIGR regression model is formed based on the MCPGIG distribution.

In the MCPGIGR regression framework, the modified Bessel function of the third kind $K_{\gamma }\left(\omega \right)$ appears naturally in the likelihood formulation. Its derivatives are computed using the standard identity $\frac{\partial K_{\gamma }\left(\varpi \right)}{\partial \varpi }=\frac{v}{\varpi }K_{\gamma }\left(\varpi \right)-K_{\gamma +1}\left(\varpi \right)$. For numerical convenience, the estimation procedure also employs the Bessel ratio $R_{\gamma }\left(\varpi \right)=\frac{K_{{}_{\gamma +1}}\left(\varpi \right)}{K_{\gamma }\left(\varpi \right)}$, which simplifies several expressions in the score function. Certain tractable forms arise when the shape parameter γ takes half-integer values (e.g., γ=−1/2,−3/2,−5/2), as discussed in [28]. The regression model

(8) $$\lambda_{j}\left(q_{j}\right)=\frac{q_{ij}\exp\left(x_{i}^{T}\beta_{j}\right)-q_{i1}\exp\left(x_{i}^{T}\beta_{1}\right)}{\xi R_{\gamma }\left(\varpi \right)}$$

when the MCPGIG distribution is specified with γ=−1/2, the regression model $\lambda_{j}\left(q_{j}\right)=\frac{\left(q_{ij}\exp\left(x_{i}^{T}\beta_{j}\right)-q_{i1}\exp\left(x_{i}^{T}\beta_{1}\right)\right)}{\xi }.$ The parameters to be estimated are $\theta_{MCPGIGR}={\left[\beta_{1}^{T}\;\beta_{2}^{T}\;\cdots \;\beta_{j}^{T}\tau \;\xi \;\gamma \right]}^{T};j=1,2,...,m$. Then, the log-likelihood function of the MCPGIG distribution that $y_{i}={\left[\begin{array}{cccc} y_{i1} & y_{i2} & ... & y_{ij} \end{array}\right]}^{T};j=1,2,...,m$ obtained is as follows:

(9) $$\ln L\left(\theta_{\text{MCPGIGR}}\right)=\ln\left(\prod_{i=1}^{n}P\left(y_{i}|\beta_{1}^{T},\beta_{2}^{T},\cdots ,\beta_{j}^{T},\tau ,\xi ,\gamma \right)\right)=\sum_{i=1}^{n}\ln P\left(y_{i}|\beta_{1}^{T},\beta_{2}^{T},\cdots ,\beta_{j}^{T},\tau ,\xi ,\gamma \right).$$

The natural logarithm of the likelihood function in Eq. (9) is obtained by applying the logarithmic transformation of regression model $\lambda_{j}\left(q_{j}\right)$ in (8) to the pmf in Eq. (6) and summing it over all observations. Differentiating this log-likelihood with respect to the model parameters $\theta_{\text{MCPGIGR}}$ yields the corresponding first-order derivatives (see **Appendix A.3)**. The first part of the log-likelihood arises from the Multivariate Poisson component, while the second part is contributed by the GIG mixing mechanism. Differentiating the log-likelihood with respect to the parameters under this specification yields the corresponding first derivatives.

The first-order derivatives of the log-likelihood (see **Appendix A.4**), do not yield closed-form solutions when set to zero, thus requiring an iterative procedure for parameter estimation. In this study, the BHHH algorithm is employed because it allows the Hessian matrix to be approximated without computing the second derivatives of the MCPGIGR likelihood [31,32]. The method relies solely on the gradient vector and the outer-product-of-gradients approximation of the Hessian, which enhances numerical stability for complex likelihood structures. The algorithm begins with an initial parameter vector and iteratively updates the estimates to maximize the likelihood function. Therefore, the BHHH method is employed utilizing the algorithm outlined in Table 1.

**Table 1:** BHHH iteration procedure.

Step	Procedure	Description
1	Initialize parameter values	Set the initial parameter vector ${{\hat{\theta }}_{\text{MCPGIGR}}}^{\left(0\right)}=\left[\begin{array}{cccc} \hat{\beta }{{}_{1}^{T}}^{\left(0\right)} & \hat{\beta }{{}_{2}^{T}}^{\left(0\right)} & ... & \hat{\beta }{{}_{j}^{T}}^{\left(0\right)}\;\begin{array}{cc} {\hat{\tau }}^{\left(0\right)} & {\hat{\xi }}^{\left(0\right)}\;\;\gamma^{\left(0\right)} \end{array} \end{array}\right]$j=1,2,...,m. The initial value of the parameter ${{\hat{\theta }}_{\text{MCPGIGR}}}^{\left(0\right)}$ is determined through separate MPR. The initial values for the overdispersion parameter τ and ξ are obtained by covariance and averaging the response variable observed overdispersion based on the variance of MCPGIGR [33].
2	Compute gradient vector	Evaluate the gradient vector, $g({\hat{\theta }}_{\text{MCPGIGR}}^{\left(t\right)})$ where each elements represents the first derivative of the log-likelihood function served in **Appendix A.4**.
3	Compute Hessian matrix	Construct the observed information matrix: $H\left({\hat{\theta }}_{\text{MCPGIGR}}^{\left(t\right)}\right)=-\sum_{i=1}^{n}g_{i}\left({\hat{\theta }}_{\text{MCPGIGR}}^{\left(t\right)}\right)g_{i}{\left({\hat{\theta }}_{\text{MCPGIGR}}^{\left(t\right)}\right)}^{T}$
4	Update parameter estimates	Update parameter values that iteration starts from *t* = 0 : ${\hat{\theta }}_{\text{MCPGIGR}}^{\left(t+1\right)}={\hat{\theta }}_{\text{MCPGIGR}}^{\left(t\right)}-H^{-1}\left({\hat{\theta }}_{\text{MCPGIGR}}^{\left(t\right)}\right)g\left({\hat{\theta }}_{\text{MCPGIGR}}^{\left(t\right)}\right)$, where ${\hat{\theta }}_{\text{MCPGIGR}}^{\left(t\right)}$ is the parameter vector in the t-th iteration.
5	Check convergence	Repeat step (2–4) for t=t+1. The iteration will stop whe $∥{\hat{\theta }}_{\text{MCPGIGR}}^{\left(t+1\right)}-{\hat{\theta }}_{\text{MCPGIGR}}^{\left(t\right)}∥\leq \varepsilon$, where $\varepsilon =10^{-7}$
6	Covariance	Get $SE\left({\hat{\beta }}_{jk}\right)=\sqrt{\hat{\text{var}}\left({\hat{\beta }}_{jk}\right)}$. The values of $\hat{\text{var}}\left({\hat{\beta }}_{jk}\right)$ is obtained from the main diagonal elements of the covariance matrix of the equation $Cov\left({\hat{\theta }}_{\text{MCPGIGR}}\right)=-\hat{E}\left(H^{-1}\left({\hat{\theta }}_{\text{MCPGIGR}}\right)\right)\underset{n\to \infty }{\approx }-H^{-1}\left({\hat{\theta }}_{\text{MCPGIGR}}\right)$.

The BHHH method utilizes the outer product of the individual gradients (score vectors) to approximate the Hessian matrix in each iteration, which enhances numerical stability and computational efficiency. The BHHH algorithm updates parameters using the outer product of individual score vectors, thereby obviating the need to compute the exact Hessian. In practice, only per-observation scores are required; a line search is employed to ensure ascent, and convergence is checked using standard criteria. Asymptotic standard errors are obtained from the inverse of the accumulated outer-product-of-gradients matrix.

### Hypothesis testing procedures for the mcpgigr model

We use MLRT for simultaneous hypotheses and Wald Z tests for partial (coefficient-wise) inference.

**Simultaneous Tests.** The significance of the regression parameters and the following hypothesis are determined through simultaneous hypothesis testing.:

$H_{0}:\beta_{j1}\;=\;\beta_{j2}\;=\;...\;=\;\beta_{\text{jp}}=0;j=1,2,...,m$

$H_{1}:\text{at}\text{least}\text{one}\beta_{jk}\neq 0,\text{with}k=1,2,...,p\text{and}j=1,2,...,m$.

Let $\Omega_{\text{MCPGIGR}}=\left\{\beta_{1}^{T},\beta_{2}^{T},...,\beta_{j}^{T},\tau ,\xi ,\gamma \right\};j=1,2,...,m$ denote the full parameter vector and $\omega_{\text{MCPGIGR}}=\left\{\beta_{01\omega },\beta_{02\omega },...,\beta_{0j\omega },\tau_{\omega },\xi_{\omega },\gamma_{\omega }\right\};j=1,2,...,m$ the restricted parameter under the null hypothesis. Following this, the log-likelihood function for the full model is constructed based on the MCPGIG distribution. This log-likelihood formulation corresponds to the equation provided in Eq. (10) of the model specification section.

(10) $$\ln L\left(\Omega_{\text{MCPGIGR}}\right)=\sum_{i=1}^{n}\ln(\frac{\xi^{-\gamma }{\left(\frac{q_{i1}\exp\left(x_{i}^{T}\beta_{1}\right)}{\xi }\right)}^{y_{i1}}}{K_{\gamma }\left(\varpi \right)y_{i1}!}\prod_{j=2}^{m}\frac{{\left(\frac{q_{ij}\exp\left(x_{i}^{T}\beta_{j}\right)-q_{i1}\exp\left(x_{i}^{T}\beta_{1}\right)}{\xi R_{\gamma }\left(\varpi \right)}\right)}^{y_{ij}-y_{i1}}}{\left(y_{ij}-y_{i1}\right)!}{\left(\sqrt{\frac{\varpi \xi^{2}}{\psi^{*}}}\right)}^{\left(y_{ij}+\gamma \right)}K_{{}_{y_{ij}+\gamma }}\left(\sqrt{\varpi \psi^{*}}\right))$$

where $\varpi =\sqrt{\tau^{2}+\xi^{2}}-\xi ;\psi^{*}=\left(2q_{ij}\sum_{j=2}^{m}\frac{\exp(x_{i}^{T}\beta_{j})}{R_{\gamma }\left(\varpi \right)}+\varpi \right)$. Similarly, a log-likelihood function is derived for the parameter set under the null hypothesis, which may include fewer or constrained parameters depending on the hypothesis tested provided in Eq. (11) *.*

(11) $$\ln L\left(\omega_{\text{MCPGIGR}}\right)=\sum_{i=1}^{n}\ln(\frac{{\xi_{\omega }}^{-\gamma_{\omega }}{\left(\frac{q_{i1}\exp\left(\beta_{01}\right)}{\xi_{\omega }}\right)}^{y_{i1}}}{K_{\gamma_{\omega }}\left(\varpi_{\omega }\right)y_{i1}!}\prod_{j=2}^{m}\frac{{\left(\frac{q_{ij}\exp\left(\beta_{0j}\right)-q_{i1}\exp\left(\beta_{01}\right)}{\xi_{\omega }R_{\gamma_{\omega }}\left(\varpi \right)}\right)}^{y_{ij}-y_{i1}}}{\left(y_{ij}-y_{i1}\right)!}{\left(\sqrt{\frac{\varpi {\xi_{\omega }}^{2}}{{\psi_{\omega }}^{*}}}\right)}^{\left(y_{ij}+\gamma_{\omega }\right)}K_{{}_{y_{ij}+\gamma_{\omega }}}\left(\sqrt{\varpi_{\omega }{\psi_{\omega }}^{*}}\right))$$

with $\varpi_{\omega }=\sqrt{{\tau_{\omega }}^{2}+{\xi_{\omega }}^{2}}-\xi_{\omega }$ and ${\psi_{\omega }}^{*}=2\sum_{j=2}^{m}\frac{q_{ij}\exp\left(\beta_{0j\omega }\right)}{R_{\gamma_{\omega }}\left(\varpi \right)}+\varpi_{\omega }$. To proceed with the likelihood ratio test, the maximum values of the log-likelihood function under both the full and restricted models are computed. These are denoted respectively as $\ln L({\hat{\Omega }}_{\text{MCPGIGR}})$ and $\ln L({\hat{\omega }}_{\text{MCPGIGR}})$ Both estimations are obtained through MLE using the BHHH iterative algorithm as outlined in Table 1. The resulting estimates, ${\hat{\theta }}_{\Omega_{\text{MCPGIGR}}}$ and ${\hat{\theta }}_{\omega_{\text{MCPGIGR}}}$, represent the maximum likelihood estimators of the parameter vectors under the full model and under the null hypothesis, respectively. The estimators for the parameters under $H_{0}$_,_ is ${\hat{\theta }}_{\omega_{\text{MCPGIGR}}}$ further, the odds ratio is determined as follows: $\Lambda_{\text{MCPGIGR}}=\frac{L({\hat{\omega }}_{\text{MCPGIGR}})}{L({\hat{\Omega }}_{\text{MCPGIGR}})}<\Lambda_{0},\text{dimana}0<\Lambda_{0}<1$. This equation is equivalent to: ${\left(\Lambda_{\text{MCPGIGR}}\right)}^{-2}={\left(\frac{L({\hat{\omega }}_{\text{MCPGIGR}})}{L({\hat{\Omega }}_{\text{MCPGIGR}})}\right)}^{-2}$, with

(12) $$G_{\text{MCPGIGR}}^{2}={\left(\Lambda_{\text{MCPGIGR}}\right)}^{-2}=2\left(\ln L\left({\hat{\Omega }}_{\text{MCPGIGR}}\right)-\ln L\left({\hat{\omega }}_{\text{MCPGIGR}}\right)\right)$$

The Eq. (12) continues determine the asymptotic distribution of the test statistic $G_{MCPGIGR}^{2}$. Then $G_{MCPGIGR}^{2}\overset{d}{\to }\chi_{pm}^{2}$ where pm is the number of degree freedom such as the number parameters in vector ${\hat{\beta }}_{j}$. The rejection region for the null hypothesis $H_{0}$​, based on the quantiles of the $\chi_{pm}^{2}$ distribution with pm degrees of freedom.

**Partial tests.** If the simultaneous hypothesis test rejects H_0_, then testing continues with a partial test to determine which predictor variables individually influence the response variable. The hypothesis for the partial test is as follows:

$\begin{array}{c} H_{0}:\beta_{jk}=0\\ H_{1}:\beta_{jk}\neq 0,\text{with}k=1,2,..,p\text{and}j=1,2,...,m. \end{array}$

The asymptotic normality of the MLE is not derived in detail here but follows from the general asymptotic theory of MLE under standard regularity conditions [34]. The test statistic $Z_{\text{MCPGIGR}}$ is the statistic asymptotically standard normal. Thus, The test statistic used is:

(13) $$Z_{\text{MCPGIGR}}=\frac{{\hat{\beta }}_{jk}}{SE({\hat{\beta }}_{jk})}\underset{n\to \infty }{∼}N\left(0,1\right)$$

The rejection region for $H_{0}$ is when the $|Z_{\text{MCPGIGR}}|>Z_{\alpha /2}$ where α is the significance level. imultaneous hypotheses are evaluated using a likelihood-ratio test (comparing the unrestricted and restricted models), whereas individual predictor effects are assessed with Wald Z-statistics based on the asymptotic MLE variance (from the outer product of gradients or the observed Hessian). Together, these procedures provide a coherent testing framework for multivariate responses.

#### Implementation note

**Fig. 1:**
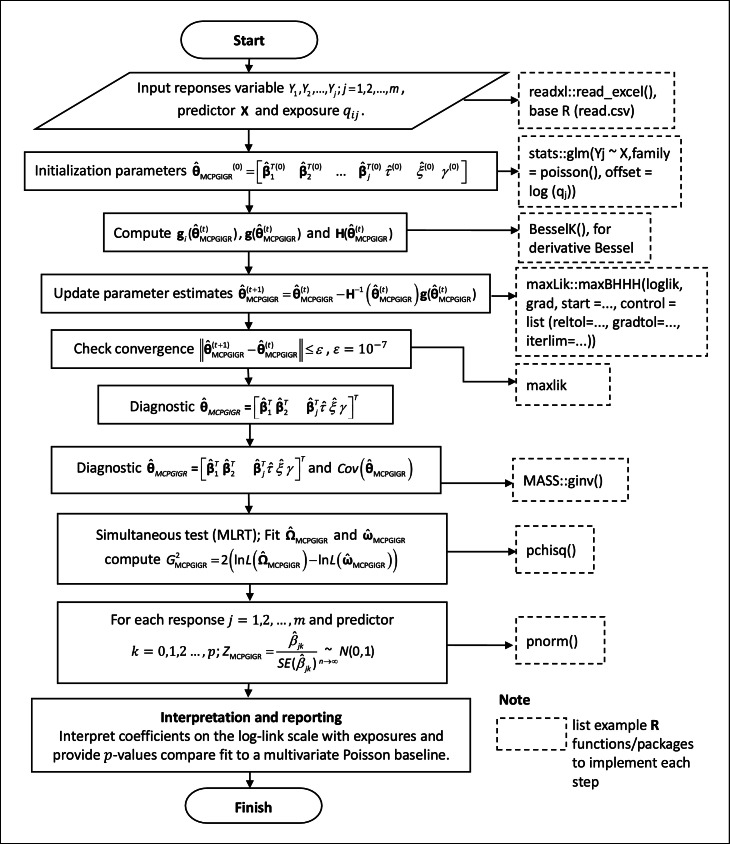
Workflow for the MCPGIGR application.

As an implementation note following the workflow in Fig. 1, the entire procedure can be replicated in R, in this research was using R 4.3.3 version, using the following core packages: maxLik for BHHH/OPG optimization; base stats functions—most notably glm() to initialize Poisson models with offset = log(q), together with pnorm(), pchisq(), qchisq(),and besselK(); MASS for matrix inversion (to obtain covariance); and readxl for importing .xlsx files. For numerical stability with extreme Bessel arguments or high-precision arithmetic, Bessel (robust evaluation of the modified Bessel function). For benchmarking against alternative count models or a baseline, COUNT, gamlss, and MixedPoisson are useful.

In practice, the BHHH-based MLE routine is sensitive to the choice of starting values, particularly for the GIG shape and scale parameters. We recommend initializing the Poisson components via glm() and obtaining initial GIG parameters using simple method-of-moments heuristics, while monitoring convergence through changes in the log-likelihood and the gradient norm. Each BHHH iteration requires a full pass over the data to compute the log-likelihood and score, so the computational cost scales approximately linearly with the number of observations and parameters. For datasets of similar size to our empirical application (tens of areas and a few hundred observations), convergence is typically achieved within a reasonable time on a standard desktop machine, whereas larger and higher-dimensional multivariate count datasets will naturally entail longer runtimes. In such settings, users may benefit from employing compiled code for Bessel function evaluations, parallelizing score computations, or using stable implementations such as lgamma()-based factorial calculations to keep computation manageable.

## Empirical results

We present two complementary pieces of evidence: a simulation study (Sections 3.1–3.2) and an applied case study (Section 3.3). The simulation is tailored to MCPGIGR and evaluates whether the proposed model and its MLE estimation procedure perform well in finite samples under controlled conditions. We vary sample size and the half-integer shape parameter γ, generate data from the MCPGIG distribution with known coefficients, and assess estimator bias/variance, and model fit (AICc). We then apply MCPGIGR to maternal and neonatal mortality data from East Java (2023), interpret the effects under the log-link with exposures, and compare goodness-of-fit against a multivariate Poisson baseline.

### Simulation design

To assess whether the proposed model performs well under the developed estimation procedure, we conduct a simulation tailored to the MCPGIGR framework in a trivariate response setting with three predictors. The simulation compares MPR and MCPGIGR with three different options of shape parameter (γ), i.e., −1/2,−3/2,−5/2 value. To simulate data, it is necessary to generate synthetic datasets that reflect the relationship between predictor and response variables, with the response following the MP and MCPGIG distribution. The simulation procedure consists of the following steps:

### Simulation result

In this section, we present the results of a simulation study conducted to evaluate the performance of the MCPGIGR model. Parameter estimation was performed using the MLE procedure, followed by the BHHH algorithm as outlined in Table 1. The estimation results include a comparison between the true parameter values and the average estimated values obtained from the simulation, as presented inTable 3. This table displays the estimates for each parameter under different configurations of the shape parameter γ and across varying sample sizes.

**Table 2:** Simulation design for MCPGIGR (predictors, shape γ, sample sizes, replications).

Steps
1. Define the true values of the regression parameters $\beta_{1}=\left[-5\;-0.05\;0.4\right]$ and $\beta_{2}=\left[1\;0.07\;0.05\right]$.
2. Generate predictor $X_{1},X_{2}∼U\left(0,10\right)$; set n∈{100,300} replicated 500 times.
3. Set dispersion/scale :τ=0.5 and ξ=1, see [33]
4. Simulate response variable data from the Multivariate Poisson and MCPGIG, see Eqs. (3) and (6).
5. Estimate parameters via MPR and MCPGIGR (BHHH).
6. Compute $Bias\left({\hat{\theta }}_{\text{MCPGIGR}}\right)={\bar{\hat{\theta }}}_{\text{MCPGIGR}}-\theta_{true}$, $\text{Var}({\hat{\theta }}_{\text{MCPGIGR}})$ (see in Table 2 step 6) and Akaike Information Criterion $AIC_{c}=-2\ln L\left({\hat{\theta }}_{\text{MCPGIGR}}\right)+2p+\frac{2p(p+1)}{n-p-1}$ , of the estimated parameters to assess estimator performance, with $L({\hat{\theta }}_{\text{MCPGIGR}})$ is the maximized likelihood function evaluated at the estimated parameters ${\hat{\theta }}_{\text{MCPGIGR}}$, p=2, p is number of predictor parameters in the model, n is sample size .
7. Summarize results; compare across γ∈{−1/2,−3/2,−5/2}.

**Table 3:** True parameters and average estimates across replications for MCPGIGR.

Parameter	True	Sampel/Shape					
		n=100			n=300		
		γ			γ		
		−1/2	−3/2	−5/2	−1/2	−3/2	−5/2
$\beta_{01}$	−5.000	−5.261	−6.225	−5.257	−5.029	−7.424	−5.453
$\beta_{11}$	−0.050	−0.049	−0.048	−0.037	−0.049	−0.051	−0.048
$\beta_{21}$	0.400	0.396	0.394	0.427	0.400	0.400	0.401
$\beta_{02}$	1.000	0.727	0.265	0.567	0.975	1.369	0.571
$\beta_{12}$	0.070	0.064	0.053	0.061	0.069	0.045	0.061
$\beta_{22}$	0.050	0.056	0.062	0.057	0.050	0.074	0.056
τ	0.500	0.269	0.235	0.583	0.504	0.544	0.562
ξ	1.000	0.361	0.607	0.831	0.974	0.020	0.848

**Fig. 2:**
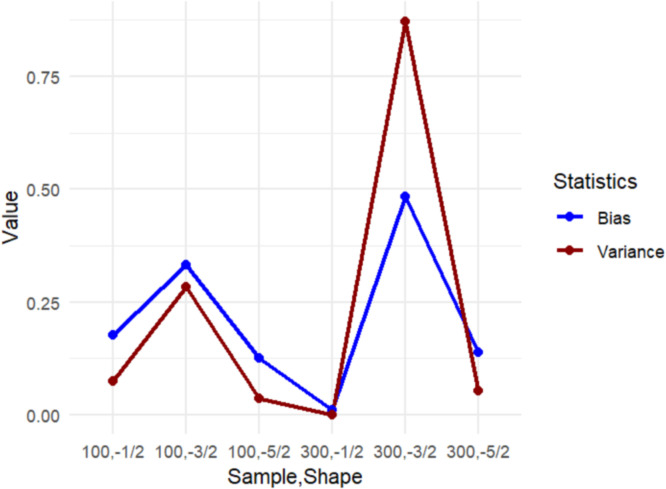
Mean absolute bias and variance of estimators by shape parameter γ and sample size.

Further insights are provided by Fig. 2, which illustrates the mean absolute bias and variance across scenarios corresponding to those reported in Table 3. The graphical representation summarizes the differences in bias and variance across various shape parameter configurations. Based on the results presented in Table 3 and Fig. 2, it is clear that the MCPGIGR model demonstrates relatively accurate parameter estimates, particularly when sample sizes are larger (i.e., n=300). This finding suggests that parameter estimates become more stable and consistent with an increase in sample size. The variance tend to decrease with a larger sample size, indicating an improvement in the model's precision and accuracy.

In terms of estimator performance, the simulation results show that the MCPGIGR estimates exhibit small bias for moderate to large sample sizes, with bias approaching zero as n increases, indicating consistency of the MLE under different shape parameters γ. The variance of the estimators decreases systematically with larger sample sizes, reflecting improved stability of the BHHH updates. Across the three shape settings γ=−1/2,−3/2, and −5/2, the model remains numerically stable, although heavier-tailed cases (e.g., γ=−5/2) produce slightly larger variability for small samples. No convergence failures were observed, and the log-likelihood increased monotonically across iterations. These diagnostics collectively confirm that the MCPGIGR estimators perform reliably and are robust across different distributional configurations.

Additionally, the simulation involved the calculation of the Corrected Akaike Information Criterion (AICc) for the three shape parameter configurations, with a sample size of n=300. The AICc values were derived from the likelihood function in Eq. (8), obtained through the MCPGIGR modeling process, and the results are presented in Table 4.

**Table 4:** AICc comparison for MPR and MCPGIGR.

Model	Shape	AICc (*n* = 300)
MPR	-	2378.63
MCPGIGR	−1/2	1924.60
	−3/2	2280.80
	−5/2	2234.50

The comparison of AICc values between MCPGIGR and the MPR model (Table 4) further indicates that MCPGIGR yields a lower AICc, suggesting that it provides a better model fit compared to the baseline model. This result supports the use of MCPGIGR as a more flexible and accurate model for handling overdispersed data with response dependencies. Several studies have compared families of mixed Poisson models. The research [35] show that the shape choice γ=−1/2 serves as a strong analytical baseline. Nevertheless, in an empirical application to the Local Government Property Insurance Fund (LGPIF) data, the specification with γ=−3/4 achieves superior model fit relative to more conventional mixed Poisson formulations lacking the additional flexibility of the GIG family. In a related direction, [36] analyze a Bivariate Poisson Generalized Inverse Gaussian (BPGIG) model and demonstrate its effectiveness in addressing overdispersion in bivariate count data. Overall, these simulation results demonstrate that the proposed estimation procedure for the MCPGIGR model provides accurate and reliable parameter estimates, even under varying parameter settings, thereby ensuring its applicability for empirical studies.

### Application

As an illustrative case study, the MCPGIGR model was applied to maternal and neonatal mortality data from East Java Province in 2023, covering 38 districts/cities and two response variables. N Maternal mortality (Y_1_) is defined as the death of a woman during pregnancy or within 42 days of pregnancy termination from causes related to pregnancy or its management [37]. The mean maternal mortality rate was 12.97 with a coefficient of variation (CV) of 78.86 %. Neonatal mortality (Y_2_), defined as the death of an infant within the first 28 days of life, had a mean of 89.53 and a CV of 73.74 %. The high CV values indicate substantial overdispersion, motivating the use of the MCPGIG distribution with the shape parameter fixed at −1/2. All R scripts, data files, and a fully reproducible HTML report for the MCPGIGR analysis are publicly available at the following links:

• GitHub repository: https://github.com/yusriantihanike/MCPGIGR_Method

• RPubs report: http://rpubs.com/yusriantihanike/1371097

These resources provide the complete reproducible workflow for the empirical analysis, including data preprocessing, model specification, estimation routines, diagnostic procedures, and output tables. The model components and estimation steps can be rerun directly using the provided scripts.

Six predictors were included in the analysis: Percentage of Tablet Consumption for Pregnant Women (X_1_), Percentage of Managed Obstetric Complications (X_2_), Percentage of Family planning participants (X_3_), the percentage of Antenatal care visits (X_4_), Percentage of healthcare professionals (X_5_), and Percentage of proper sanitation (X_6_). Exposure was defined as the number of pregnant women for maternal mortality (Y_1_) and the number of live births for neonatal mortality (Y_2_) in each district/city. Table 5, Table 6 summarize the descriptive statistics for both the predictors and responses.

**Table 5:** Summary of predictor variables.

Variable	Mean (Standard Deviation)
Percentage of Tablet Consumption for Pregnant Women (X_1_)	81.65 (14.76)
Percentage of Managed Obstetric Complications (X_2_)	20.59 (4.40)
Percentage of Family planning participants (X_3_)	59.88 (16.59)
Percentage of Antenatal care visits (X_4_)	78.04 (11.10)
Percentage of healthcare professionals (X_5_)	20.44 (15.10)
Percentage of proper sanitation (X_6_)	92.35 (7.99)

Source. Health Profile East Java, 2023.

**Table 6:** Summary statistics for maternal and neonatal mortality (responses).

Variable	Mean	Variance	Coefficient of Variation	Min	Max
Maternal Mortality Rate (Y_1_)	12.97	104.67	78.86	0	50
Neonatal Mortality Rate (Y_2_)	89.53	4358.63	73.74	5	287

Source. Health Profile East Java, 2023.

**Fig. 3:**
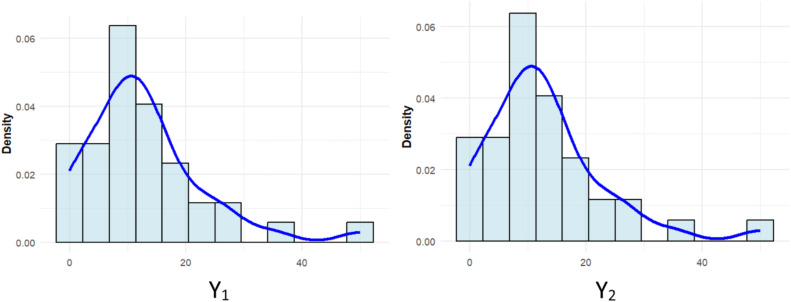
Histograms of maternal (Y₁) and neonatal (Y₂) mortality across districts/cities.

Before estimation, the suitability of the MCPGIG distribution was assessed using Crockett’s test [38]. The test statistic was 0.0786 with a p-value of 0.693, indicating no evidence against the MCPGIG assumption at the 5 % significance level. Histograms of the two response variables are presented in Fig. 3.

Initial values for parameter estimation are provided in Table 7. . Regression coefficients were initialized using standard Poisson regression to obtain stable starting values for the mean structure. The scale parameter ξ was set to 1, while the dispersion parameter τ was initialized at 0.5448 based on the relative excess variance across both responses [33].

**Table 7:** Initial parameter values for MCPGIGR estimation.

Parameter	Response						
	$\beta_{0}$	$\beta_{1}$	$\beta_{2}$	$\beta_{3}$	$\beta_{4}$	$\beta_{5}$	$\beta_{6}$
*Y_1_*	−7.2905	−0.0035	0.0267	0.0036	−0.0103	−0.0288	0.0103
*Y_2_*	−4.0517	0.0026	−0.0149	0.0025	−0.0287	0.0071	0.0098

Table 8 presents the parameter estimation results for the MCPGIGR model with two responses and six predictors. For maternal mortality, antenatal care (X₄) and healthcare professionals (X₅) were significant predictors, consistent with their roles in early detection and timely management of pregnancy complications. For neonatal mortality, all six predictors were statistically significant, indicating that maternal nutrition, complication management, antenatal visits, family planning, healthcare availability, and sanitation jointly contribute to neonatal survival. The model of MCPGIGR stated by :

$$\begin{array}{c} \hat{E(y_{1})}=\exp\left(-4.9212-0.0034x_{1}+0.0064x_{2}+0.0003x_{3}-0.0168x_{4}-0.0121x_{5}+0.0012x_{6}\right)\\ \hat{E(y_{2})}=\exp\left(-4.2743+0.0044x_{1}-0.0166x_{2}+0.0039x_{3}-0.0311x_{4}+0.0108x_{5}+0.0157x_{6}\right) \end{array}$$

**Table 8:** MCPGIGR parameter estimates, SE, Z-statistics, and p-values.

Parameter	*Y_1_*			
	Estimate	SE	Z	p-Value
$\beta_{01}$	−4.9212	0.0165	−297.9419	0.0000*
$\beta_{11}$	−0.0034	0.0020	−1.6751	0.0938
$\beta_{21}$	0.0064	0.0032	1.9601	0.0499
$\beta_{31}$	0.0003	0.0009	0.4014	0.6880
$\beta_{41}$	−0.0168	0.0025	−6.5917	0.0000*
$\beta_{51}$	−0.0121	0.0031	−3.8009	0.0001*
$\beta_{61}$	0.0012	0.0028	0.4524	0.6509
**Parameter**	***Y_2_***			
	**Estimate**	**SE**	**Z**	**p-Value**
$\beta_{02}$	−4.2743	0.0335	−127.3982	0.0000*
$\beta_{12}$	0.0044	0.0010	4.1063	0.0000*
$\beta_{22}$	−0.0166	0.0022	−7.5059	0.0000*
$\beta_{32}$	0.0039	0.0004	9.4428	0.0000*
$\beta_{42}$	−0.0311	0.0010	−29.9436	0.0000*
$\beta_{52}$	0.0108	0.0009	11.1926	0.0000*
$\beta_{62}$	0.0157	0.0014	10.8288	0.0000*

⁎ Significant at α = 5 %.

The likelihood ratio test statistic was conducted to evaluate the overall significance of the model. The test statistic was 336.7596, which exceeded the chi-square critical value of 21.026 (df = 12, α = 0.05), leading to rejection of the null hypothesis and confirming that the predictors jointly influence the response variables. The estimated dispersion parameters were τ = 1.2975 (SE = 0.0153) and ξ = 0.04845 (SE = 0.0083). In the empirical application, the MCPGIGR model provides a substantially improved fit compared with the baseline multivariate Poisson specification, as evidenced by an approximate 19 % reduction in AICc. Beyond this improvement in information criteria, additional diagnostics indicate that the model successfully captures both marginal overdispersion and cross-response dependence, as reflected in the mixing parameters τ and ξ, which are significantly. The estimation procedure also exhibits strong numerical stability: the BHHH algorithm converges in fewer than 30 iterations, and the outer-product-of-gradients matrix remains positive definite throughout the optimization process. Taken together, these findings demonstrate that MCPGIGR offers a more flexible and empirically appropriate representation of the maternal and neonatal mortality data than the standard multivariate Poisson model.

These findings are consistent with prior studies emphasizing the importance of healthcare access, maternal education, sanitation, and clinical care in reducing maternal and neonatal mortality in Indonesia [39,40]. Previous work highlights that proximity to healthcare facilities, the quality of primary health services, family planning programs, and maternal care practices significantly influence mortality outcomes. Similarly, studies on neonatal outcomes underscore the roles of antenatal care, skilled health personnel, and maternal health conditions in determining neonatal survival. The MCPGIGR model aligns with these observations and provides a more flexible multivariate framework for jointly modeling maternal and neonatal mortality.

Although this article focuses on maternal and neonatal mortality in East Java, the MCPGIGR framework can be applied to a wide range of multivariate count-data problems. In insurance and actuarial science, the model can be used to analyze joint claim counts of different claim types [41], particularly when both overdispersion and dependence across claim types are present. In transportation and traffic safety, the model can accommodate correlated counts of different crash types while simultaneously capturing marginal overdispersion and common-shock dependence induced by shared risk factors [42]. More broadly, any setting involving overdispersed and correlated count responses observed on the same statistical units (such as regions, hospitals, road segments, or customers) stands to benefit from this modeling framework.

## Ethics statements

This study utilizes secondary data obtained from publicly available publications of the East Java Provincial Health Profile. No human subjects were directly involved in the research, and no individual-level or personally identifiable information was used. The dataset is available upon reasonable request to the corresponding author.

## Limitations

1. The construction of hypothesis testing procedures (e.g., likelihood ratio test and Wald test) in this study relies on asymptotic theory under standard regularity conditions. A complete theoretical derivation tailored to the MCPGIGR framework is not provided, and the results are primarily based on general asymptotic properties of MLE.

2. The choice of predictor variables was made by considering both the theoretical framework and the availability of data. Consequently, potentially relevant covariates not included in the dataset could not be incorporated into the model.

## Related research article

Mardalena, S., Purhadi, P., Purnomo, J.D.T., Prastyo, D.D. (2020), Parameter estimation and hypothesis testing of multivariate Poisson inverse Gaussian regression, https://www.mdpi.com/2073–8994/12/10/1738.

## For a published article

none

## CRediT authorship contribution statement

**Yusrianti Hanike:** Conceptualization, Methodology, Software, Writing – original draft, Visualization. **Purhadi:** Conceptualization, Methodology, Writing – review & editing, Validation, Supervision. **Achmad Choiruddin:** Conceptualization, Methodology, Writing – review & editing, Validation, Supervision.

## Declaration of competing interest

The authors declare that they have no known financial interests or personal relationships that could have appeared to influence the work reported in this article.

## Figures and Tables

**Table:** 

AICc	Corrected Akaike Information Criterion
BHHH	Berndt–Hall–Hall–Hausman
BPGIG	Bivariate Poisson Generalized Inverse Gaussian
LBW	Low Birth Weight
MCPGIG	Multivariate Correlated Poisson Generalized Inverse Gaussian
MCPGIGR	Multivariate Correlated Poisson Generalized Inverse Gaussian Regression
MLE	Maximum Likelihood Estimation
MLRT	Maximum Likelihood Ratio Test
MPR	Multivariate Poisson Regression
PGIG	Poisson Generalized Inverse Gaussian
pmf	Probability Mass Function
SE	Standard Error

## Data Availability

Data will be made available on request.

## Appendix A. Mathematical Details

**A.1. Multivariate Poisson distribution**

The construction of the Multivariate Poisson Distribution began with the definition of response variable $Y_{1},Y_{2},...,Y_{j};j=1,2,...,m$ as follows in Eq. (2) and the probability generating function (PGF) can be derived as follows:

(A.1.1) $$G\left(t_{1},t_{2},...,t_{j}\right)=E\left(t_{1}^{Y_{1}}t_{2}^{Y_{2}}...t_{j}^{Y_{j}}\right)=E\left(t_{2}^{Z_{2}}...t_{j}^{Z_{j}}{\left(t_{1}t_{2}...t_{j}\right)}^{Z_{1}}\right).$$

Given that the random variables $Z_{1},Z_{2},...,Z_{j};j=1,2,...,m$ are mutually independent and follow Poisson distributions in Eq. (1), the PGF expressed by

(A.1.2) $$G\left(t_{1},t_{2},...,t_{j}\right)=\exp\left(-\left(\lambda_{1}\left(q_{1}\right)+\lambda_{2}\left(q_{2}\right)+...+\lambda_{j}\left(q_{j}\right)\right)\right)\sum_{z_{2}=0}^{\infty }...\sum_{z_{j}=0}^{\infty }\sum_{z_{1}=0}^{\infty }\frac{{\left(\lambda_{2}\left(q_{2}\right)\right)}^{z_{2}}\ldots {\left(\lambda_{j}\left(q_{j}\right)\right)}^{z_{j}}{\left(\lambda_{1}\left(q_{1}\right)\right)}^{z_{1}}}{z_{2}!...z_{j}!z_{1}!}{t_{1}}^{z_{1}}{t_{2}}^{z_{1}+z_{2}}...{t_{j}}^{z_{1}+z_{j}}$$

We assume a transformation using Eq. (2) is given by $y_{1}=z_{1},y_{j}=z_{j}+z_{1},\;j=2,...,m,\;$ and $\lambda_{j}\left(q_{j}\right)\geq 0$, then PGF is given by

(A.1.3) $$G\left(t_{1},t_{2},...,t_{j}\right)=\exp\left(-\left(\lambda_{1}\left(q_{1}\right)+\sum_{j=2}^{m}\lambda_{j}\left(q_{j}\right)\right)\right)\sum_{y_{2}=0}^{\infty }...\sum_{y_{j}=0}^{\infty }\sum_{y_{1}=0}^{\infty }\frac{{\left(\lambda_{1}\left(q_{1}\right)\right)}^{y_{1}}}{y_{1}!}{t_{1}}^{y_{1}}\prod_{j=2}^{m}\frac{{\left(\lambda_{j}\left(q_{j}\right)\right)}^{y_{j}-y_{1}}}{\left(y_{j}-y_{1}\right)!}{t_{2}}^{y_{2}}...{t_{j}}^{y_{j}}$$

Based on equation (A.1.3), the joint pmf of the Multivariate Poisson distribution can be expressed in Eq. (3).

**A.2. MCPGIG Distribution**

The construction of the MCPGIG Distribution resulting from an error term $\varepsilon_{j}$ is added to $\lambda_{j}$ in Eq. (4). Therefore, $Y_{1},Y_{2},...,Y_{m}$ follows a mixed Poisson distribution based on (4), (3), its integral pmf follows

(A.2.1) $$P\left(y|\lambda \left(q\right),\tau ,\gamma ,\nu \right)=\int_{0}^{\infty }e^{-\left(\lambda_{1}\left(q_{1}\right)\nu +\sum_{j=2}^{m}\lambda_{j}\left(q_{j}\right)\nu \right)}\frac{{\left(\lambda_{1}\left(q_{1}\right)\nu \right)}^{y_{1}}}{y_{1}!}\prod_{j=2}^{m}\frac{{\left(\lambda_{j}\left(q_{j}\right)\nu \right)}^{y_{j}-y_{1}}}{\left(y_{j}-y_{1}\right)!}g\left(\nu \right)dv$$

the g(ν;τ,ξ,γ) in defined in Eq. (5), then the joint probability distribution function for the MCPGIG distribution is given distribution of ν in Eq. (5) by follows:

(A.2.2) $$\begin{array}{c} P\left(y|\lambda \left(q\right),\tau ,\gamma ,\nu \right)=\int_{0}^{\infty }\prod_{j=2}^{m}\nu^{y_{j}+\gamma -1}\exp(\left(\frac{\left(-2\xi \left(\lambda_{1}\left(q_{1}\right)+\sum_{j=2}^{m}\lambda_{j}\left(q_{j}\right)\right)-\left(\sqrt{\tau^{2}+\xi^{2}}-\xi \right)\right)}{2\xi }\right)\nu \\ -\left(\frac{\left(\sqrt{\tau^{2}+\xi^{2}}-\xi \right)\xi }{2}\right)\nu^{-1})d\nu \frac{\xi^{-\gamma }{\left(\lambda_{1}\left(q_{1}\right)\right)}^{y_{1}}}{2K_{\gamma }\left(\sqrt{\tau^{2}+\xi^{2}}-\xi \right)y_{1}!}\prod_{j=2}^{m}\frac{{\left(\lambda_{j}\left(q_{j}\right)\right)}^{y_{j}-y_{1}}}{\left(y_{j}-y_{1}\right)!} \end{array}$$

Based on the integral table by [29] as mentioned in [30], it is stated that:

(A.2.3) $$\int_{0}^{\infty }\nu^{s-1}\exp\left(a\nu^{h}-b\nu^{-h}\right)d\nu =\left(\frac{2}{h}\right){\left(\frac{b}{a}\right)}^{\frac{s}{2h}}K_{\frac{s}{h}}\left(2\sqrt{ab}\right)$$

with $h=1;s=y_{j}+\gamma ;a=\frac{2\xi \left(\lambda_{1}\left(q_{1}\right)+\sum_{j=2}^{m}\lambda_{j}\left(q_{j}\right)\right)+\left(\sqrt{\tau^{2}+\xi^{2}}-\xi \right)}{2\xi };b=\frac{(\sqrt{\tau^{2}+\xi^{2}}-\xi )\xi }{2}$ the result of MCPGIG distribution according in equation A.2.2 and A.2.3 can be stated on Eq. (6) with correlation $Corr\left(Y_{j,}Y_{l}\right)={\left(\left(1+\frac{1}{\left(\lambda_{j}\left(q_{j}\right)+\lambda_{1}\left(q_{1}\right)\right)\psi_{\gamma }}\right)\left(1+\frac{1}{\left(\lambda_{l}\left(q_{l}\right)+\lambda_{1}\left(q_{1}\right)\right)\psi_{\gamma }}\right)\right)}^{\gamma }$$\psi_{\gamma }=R_{\gamma }\left(\varpi \right)-1;$$R_{\gamma }\left(\varpi \right)=\frac{K_{\gamma +1}\left(\varpi \right)}{K_{\gamma }\left(\varpi \right)}$.

**A.3 Log Likelihood MCPGIGR**

Let $\theta_{MCPGIGR}={\left[\beta_{1}^{T}\;\beta_{2}^{T}\;\cdots \;\beta_{j}^{T}\tau \;\xi \;\gamma \right]}^{T};j=1,2,..,m$ and $E\left(Y_{ij}\right)=q_{ij}e^{x_{i}^{T}\beta_{j}}$, the construction of MCPGIGR follows :

(A.3.1) $$\lambda_{j}\left(q_{ij}\right)=\frac{q_{ij}\exp\left(x_{i}^{T}\beta_{j}\right)-q_{i1}\exp\left(x_{i}^{T}\beta_{1}\right)}{\xi R_{\gamma }\left(\varpi \right)}$$

The equation (A.3.1) subtitution in Eq. (6) the probability function

(A.3.2) $$P\left(y_{i}|\theta_{\text{MPGIGR}}\right)=\frac{\xi^{-\gamma }{\left(\frac{q_{i1}\exp\left(x_{i}^{T}\beta_{1}\right)}{\xi R_{\gamma }\left(\varpi \right)}\right)}^{y_{i1}}}{K_{\gamma }\left(\varpi \right)y_{i1}!}\prod_{j=2}^{m}\frac{{\left(\frac{q_{ij}\exp\left(x_{i}^{T}\beta_{j}\right)-q_{i1}\exp\left(x_{i}^{T}\beta_{1}\right)}{\xi R_{\gamma }\left(\varpi \right)}\right)}^{y_{ij}-y_{i1}}}{\left(y_{ij}-y_{i1}\right)!}{\left(\frac{\varpi \xi^{2}}{\psi^{*}}\right)}^{\frac{y_{ij}+\gamma }{2}}K_{{}_{y_{ij}+\gamma }}\left(\sqrt{\psi^{*}\varpi }\right)$$

According to Eq. (9) the log likelihood can be expressed below :

(A.3.3) $$\ln L\left(\theta_{\text{MCPGIGR}}\right)=\sum_{i=1}^{n}\ln(\frac{\xi^{-\gamma }{\left(\frac{q_{i1}\exp\left(x_{i}^{T}\beta_{1}\right)}{\xi }\right)}^{y_{i1}}}{K_{\gamma }\left(\varpi \right)y_{i1}!}\prod_{j=2}^{m}\frac{{\left(\frac{q_{ij}\exp\left(x_{i}^{T}\beta_{j}\right)-q_{i1}\exp\left(x_{i}^{T}\beta_{1}\right)}{\xi R_{\gamma }\left(\varpi \right)}\right)}^{y_{ij}-y_{i1}}}{\left(y_{ij}-y_{i1}\right)!}{\left(\sqrt{\frac{\varpi \xi^{2}}{\psi^{*}}}\right)}^{\left(y_{ij}+\gamma \right)}K_{{}_{y_{ij}+\gamma }}\left(\sqrt{\varpi \psi^{*}}\right))$$

**A.4 BHHH Ingredients**

Let $\theta_{MCPGIGR}={\left[\beta_{1}^{T}\;\beta_{2}^{T}\;\cdots \;\beta_{j}^{T}\tau \;\xi \;\gamma \right]}^{T};j=1,2,...,m$ and $E\left(Y_{ij}\right)=q_{ij}e^{x_{i}^{T}\beta_{j}}$. Using the MCPGIG pmf derived in Eq. (6), the individual log-likelihood can be written as

(A.4.1) $$\begin{array}{c} \ln P\left(y_{i}|\theta_{\text{MPGIGR}}\right)=\ln(\frac{\xi^{-\gamma }{\left(\frac{q_{i1}\exp\left(x_{i}^{T}\beta_{1}\right)}{\xi }\right)}^{y_{i1}}}{K_{\gamma }\left(\varpi \right)y_{i1}!}\prod_{j=2}^{m}\frac{{\left(\frac{q_{ij}\exp\left(x_{i}^{T}\beta_{j}\right)-q_{i1}\exp\left(x_{i}^{T}\beta_{1}\right)}{\xi R_{\gamma }\left(\varpi \right)}\right)}^{y_{ij}-y_{i1}}}{\left(y_{ij}-y_{i1}\right)!}{\left(\sqrt{\frac{\varpi \xi^{2}}{\psi^{*}}}\right)}^{\left(\frac{y_{ij}+\gamma }{2}\right)}K_{{}_{y_{ij}+\gamma }}\left(\sqrt{\varpi \psi^{*}}\right))\\ =\ln(\underset{\text{Multivariate}\text{Poisson}}{\underset{︸}{\frac{{\left(q_{i1}\exp\left(x_{i}^{T}\beta_{1}\right)\right)}^{y_{i1}}}{y_{i1}!}\prod_{j=2}^{m}\frac{{\left(q_{ij}\exp\left(x_{i}^{T}\beta_{j}\right)-q_{i1}\exp\left(x_{i}^{T}\beta_{1}\right)\right)}^{y_{ij}-y_{i1}}}{\left(y_{ij}-y_{i1}\right)!}}}\underset{\text{mixing}\text{GIG}}{\underset{︸}{\frac{K_{{}_{y_{ij}+\gamma }}\left(\sqrt{\varpi \psi^{*}}\right)}{{\left(R_{\gamma }\left(\varpi \right)\right)}^{y_{ij}}K_{\gamma }\left(\varpi \right)}{\left(\sqrt{\frac{\varpi }{\psi^{*}}}\right)}^{y_{ij}+\gamma }}}) \end{array}$$

$$\varpi =\sqrt{\tau^{2}+\xi^{2}}-\xi ;\psi^{*}=\left(2q_{ij}\exp\left(x_{i}^{T}\beta_{j}\right)+\varpi \right)$$

Several explicit forms are available for half-integer orders. In particular when $\gamma =n+\frac{1}{2}$, the ratio admits a closed form as a rational function: $R_{n+\frac{1}{2}}\left(\varpi \right)=\frac{P_{n+1}\left(1/\varpi \right)}{P_{n}\left(1/\varpi \right)}$, where $P_{n}\left(\cdot \right)$ denotes a polynomial. In our simulations, we consider three half-integer shape parameters $\gamma \in \{-\frac{5}{2},-\frac{3}{2},\frac{1}{2}\}$ which yield closed-form expressions and stable derivatives for the MCPGIGR likelihood. $a)\gamma =-\frac{5}{2},\frac{K_{-\frac{3}{2}}\left(\varpi \right)}{K_{-\frac{5}{2}}\left(\varpi \right)}=R_{-\frac{5}{2}}\left(\varpi \right)=\frac{\varpi^{2}+\varpi }{\varpi^{2}+3\varpi +3}$; $b)\gamma =-\frac{3}{2},\frac{K_{-\frac{1}{2}}\left(\varpi \right)}{K_{-\frac{3}{2}}\left(\varpi \right)}=R_{-\frac{3}{2}}\left(\varpi \right)=\frac{\varpi }{\varpi +1}$$c)\gamma =-\frac{1}{2},\frac{K_{\frac{1}{2}}\left(\varpi \right)}{K_{-\frac{1}{2}}\left(\varpi \right)}=R_{-\frac{1}{2}}\left(\varpi \right)=1.$ The individual log-likelihood was used to obtain the gradien.

(A.4.2) $$g_{i}\left(\theta_{\text{MCPGIGR}}\right)=\frac{\partial \ln(\theta_{\text{MCPGIGR}})}{\partial \theta_{\text{MCPGIGR}}}=\frac{\partial \ln P\left(y_{i}|\theta_{\text{MPGIGR}}\right)}{\partial \theta_{\text{MCPGIGR}}}$$

(A.4.3) $$g^{T}\left({{\hat{\theta }}_{\text{MCPGIGR}}}^{\left(t\right)}\right)=[\frac{\partial \ln L({\hat{\theta }}_{\text{MCPGIGR}})}{\partial {\hat{\beta }}_{1}}\;\frac{\partial \ln L({\hat{\theta }}_{\text{MCPGIGR}})}{\partial {\hat{\beta }}_{2}}\;...\;\frac{\partial \ln L({\hat{\theta }}_{\text{MCPGIGR}})}{\partial {\hat{\beta }}_{j}}{\frac{\partial \ln L({\hat{\theta }}_{\text{MCPGIGR}})}{\partial \hat{\tau }}\;\frac{\partial \ln L({\hat{\theta }}_{\text{MCPGIGR}})}{\partial \hat{\xi }}\;\gamma ]}_{\hat{\theta }={\hat{\theta }}_{\left(t\right)}}$$

(A.4.4) $$g^{T}\left({{\hat{\theta }}_{\text{MCPGIGR}}}^{\left(t\right)}\right)=\sum_{i=1}^{n}g_{i}\left(\theta_{\text{MCPGIGR}}\right)$$

The natural logarithm of likelihood function is differentiated $\theta_{\text{MCPGIGR}}$ respect to the parameters resulting in the first derivatives. The process of first derivatives of $\beta_{1}$ as follows

$$\begin{array}{c} \frac{\partial \ln L(\theta_{\text{MCPGIGR}})}{\partial \beta_{1}}=\sum_{i=1}^{n}\frac{\partial \ln P\left(y_{i}|\beta_{1}^{T},\beta_{2}^{T},\cdots ,\beta_{j}^{T},\tau ,\xi ,\gamma \right)}{\partial \beta_{1}}\\ =\sum_{i=1}^{n}\frac{\partial }{\partial \beta_{1}}\ln\left(\frac{{\left(q_{i1}\exp\left(x_{i}^{T}\beta_{1}\right)\right)}^{y_{i1}}}{y_{i1}!}\prod_{j=2}^{m}\frac{{\left(q_{ij}\exp\left(x_{i}^{T}\beta_{j}\right)-q_{i1}\exp\left(x_{i}^{T}\beta_{1}\right)\right)}^{y_{ij}-y_{i1}}}{\left(y_{ij}-y_{i1}\right)!}\frac{K_{{}_{y_{ij}+\gamma }}\left(\sqrt{\varpi \psi^{*}}\right)}{{\left(R_{\gamma }\left(\varpi \right)\right)}^{y_{ij}-y_{i1}}K_{\gamma }\left(\varpi \right)}{\left(\sqrt{\frac{\varpi }{\psi^{*}}}\right)}^{y_{ij}+\gamma }\right)\\ =\sum_{i=1}^{n}\frac{\partial }{\partial \beta_{1}}(y_{i1}\ln\left(q_{i1}\exp\left(x_{i}^{T}\beta_{1}\right)\right)-\ln y_{i1}!+\sum_{j=2}^{m}\left(y_{ij}-y_{i1}\right)\ln\left(q_{ij}\exp\left(x_{i}^{T}\beta_{j}\right)-q_{i1}\exp\left(x_{i}^{T}\beta_{1}\right)\right)-\sum_{j=1}^{m}\ln\left(y_{ij}-y_{i1}\right)!\\ +\sum_{j=2}^{m}\ln K_{{}_{y_{ij}+\gamma }}\left(\sqrt{\varpi \psi^{*}}\right)-\sum_{j=2}^{m}\left(y_{ij}\right)\ln\left(R_{\gamma }\left(\varpi \right)\right)-\sum_{j=2}^{m}\ln K_{\gamma }\left(\varpi \right)+\sum_{j=2}^{m}\frac{\left(y_{ij}+\gamma \right)}{2}\ln\frac{\varpi }{\psi^{*}})\\ =\sum_{i=1}^{n}\left(\frac{\partial \left(y_{i1}\ln\left(q_{i1}\exp\left(x_{i}^{T}\beta_{1}\right)\right)\right)}{\partial \beta_{1}}+\frac{\partial \left(\sum_{j=2}^{m}\left(y_{ij}-y_{i1}\right)\ln\left(q_{ij}\exp\left(x_{i}^{T}\beta_{j}\right)-q_{i1}\exp\left(x_{i}^{T}\beta_{1}\right)\right)\right)}{\partial \beta_{1}}\right) \end{array}$$

(A.4.5) $$\frac{\partial \ln L(\theta_{\text{MCPGIGR}})}{\partial \beta_{1}}=\sum_{i=1}^{n}(y_{i1}x_{i}^{T}-\sum_{j=2}^{m}\left(y_{ij}-y_{i1}\right)\frac{q_{i1}\exp\left(x_{i}^{T}\beta_{1}\right)}{\left(q_{ij}\exp\left(x_{i}^{T}\beta_{j}\right)-q_{i1}\exp\left(x_{i}^{T}\beta_{1}\right)\right)}x_{i}^{T})$$

The process of first derivatives of $\beta_{j}$ as follows

(A.4.6) $$\begin{array}{c} \frac{\partial \ln L(\theta_{\text{MCPGIGR}})}{\partial \beta_{j}}=\sum_{i=1}^{n}\frac{\partial \ln P\left(y_{i}|\beta_{1}^{T},\beta_{2}^{T},\cdots ,\beta_{j}^{T},\tau ,\xi ,\gamma \right)}{\partial \beta_{j}}\\ =\sum_{i=1}^{n}(\frac{\partial \left(\sum_{j=2}^{m}\left(y_{ij}-y_{i1}\right)\ln\left(q_{ij}\exp\left(x_{i}^{T}\beta_{j}\right)-q_{i1}\exp\left(x_{i}^{T}\beta_{1}\right)\right)\right)}{\partial \beta_{j}}+\frac{\partial \left(\sum_{j=2}^{m}\ln K_{{}_{y_{ij}+\gamma }}\left(\sqrt{\varpi \psi^{*}}\right)\right)}{\partial \beta_{j}}-\frac{\partial \left(\sum_{j=2}^{m}\frac{\left(y_{ij}+\gamma \right)}{2}\ln\psi^{*}\right)}{\partial \beta_{j}})\\ =\sum_{i=1}^{n}(\sum_{j=2}^{m}\left(y_{ij}-y_{i1}\right)\frac{q_{ij}\exp\left(x_{i}^{T}\beta_{j}\right)}{\left(q_{ij}\exp\left(x_{i}^{T}\beta_{j}\right)-q_{i1}\exp\left(x_{i}^{T}\beta_{1}\right)\right)}x_{i}^{T}+\sum_{j=2}^{m}\frac{\left(\frac{y_{ij}+\gamma }{\sqrt{\varpi \psi^{*}}}K_{{}_{y_{ij}+\gamma }}\left(\sqrt{\varpi \psi^{*}}\right)-K_{{}_{y_{ij}+\gamma +1}}\left(\sqrt{\varpi \psi^{*}}\right)\right)}{K_{{}_{y_{ij}+\gamma }}\left(\sqrt{\varpi \psi^{*}}\right)}\\ \times \frac{\varpi q_{ij}\exp\left(x_{i}^{T}\beta_{j}\right)}{\left(\sqrt{\varpi \psi^{*}}\right)}x_{i}^{T}-\sum_{j=2}^{m}\frac{\left(y_{ij}+\gamma \right)}{2}\frac{2q_{ij}\exp\left(x_{i}^{T}\beta_{j}\right)}{\left(2q_{ij}\exp\left(x_{i}^{T}\beta_{j}\right)+\varpi \right)}x_{i}^{T})\\ =\sum_{i=1}^{n}x_{i}^{T}\left[\sum_{j=2}^{m}\left(y_{ij}-y_{i1}\right)\frac{q_{ij}\exp\left(x_{i}^{T}\beta_{j}\right)}{\left(q_{ij}\exp\left(x_{i}^{T}\beta_{j}\right)-q_{i1}\exp\left(x_{i}^{T}\beta_{1}\right)\right)}q_{ij}\exp\left(x_{i}^{T}\beta_{j}\right)\frac{\varpi }{\sqrt{\varpi \psi^{*}}}\frac{K_{{}_{y_{ij}+\gamma +1}}\left(\sqrt{\varpi \psi^{*}}\right)}{K_{{}_{y_{ij}+\gamma }}\left(\sqrt{\varpi \psi^{*}}\right)}\right] \end{array}$$

The natural logarithm of likelihood function is differentiated of τ as the dispersion parameter:

(A.4.7) $$\begin{array}{c} \frac{\partial \ln L(\theta_{\text{MCPGIGR}})}{\partial \tau }=\sum_{i=1}^{n}\frac{\partial \ln P\left(y_{i}|\beta_{1}^{T},\beta_{2}^{T},\cdots ,\beta_{j}^{T},\tau ,\xi ,\gamma \right)}{\partial \tau }\\ =\sum_{i=1}^{n}\left(\frac{\partial \left(\sum_{j=2}^{m}\ln K_{{}_{y_{ij}+\gamma }}\left(\sqrt{\varpi \psi^{*}}\right)\right)}{\partial \tau }-\frac{\partial \left(\sum_{j=2}^{m}\left(y_{ij}-y_{i1}\right)\ln\left(R_{\gamma }\left(\varpi \right)\right)\right)}{\partial \tau }-\frac{\partial \left(\sum_{j=2}^{m}lnK_{\gamma }\left(\varpi \right)\right)}{\partial \tau }+\frac{\partial \left(\sum_{j=2}^{m}\frac{\left(y_{ij}+\gamma \right)}{2}\ln\frac{\varpi }{\psi^{*}}\right)}{\partial \tau }\right)\\ =\sum_{i=1}^{n}(\sum_{j=2}^{m}\frac{\left(\frac{y_{ij}+\gamma }{\sqrt{\varpi \psi^{*}}}K_{{}_{y_{ij}+\gamma }}\left(\sqrt{\varpi \psi^{*}}\right)-K_{{}_{y_{ij}+\gamma +1}}\left(\sqrt{\varpi \psi^{*}}\right)\right)\tau \left(\psi^{*}+\varpi \right)}{2\left(\sqrt{\varpi \psi^{*}}\right)\sqrt{\tau^{2}+\xi^{2}}K_{{}_{y_{ij}+\gamma }}\left(\sqrt{\varpi \psi^{*}}\right)}-\sum_{j=2}^{m}\left(y_{ij}-y_{i1}\right)\frac{\partial \left(\ln\left(R_{\gamma }\left(\varpi \right)\right)\right)}{\partial \tau }\\ -\left(m-1\right)\left(\frac{\gamma }{\varpi }-\frac{K_{\gamma +1}\left(\varpi \right)}{K_{\gamma }\left(\varpi \right)}\right)\frac{\tau }{\sqrt{\tau^{2}+\xi^{2}}}+\sum_{j=2}^{m}\frac{\left(y_{ij}+\gamma \right)\tau \left(\psi^{*}-\varpi \right)}{2\varpi \psi^{*}\sqrt{\tau^{2}+\xi^{2}}}) \end{array}$$

with $\varpi =\sqrt{\tau^{2}+\xi^{2}}-\xi$ and $\psi^{*}=2\sum_{j=2}^{m}q_{ij}\exp\left(x_{i}^{T}\beta_{j}\right)+\varpi$, if

$$\frac{\partial \left(\ln\left(R_{-\frac{5}{2}}\left(\varpi \right)\right)\right)}{\partial \tau }=\frac{\tau \left(\frac{2\varpi +1}{\varpi^{2}+\varpi }-\frac{2\varpi +3}{\varpi^{2}+3\varpi +3}\right)}{\sqrt{\tau^{2}+\xi^{2}}};\frac{\partial \left(\ln\left(R_{-\frac{3}{2}}\left(\varpi \right)\right)\right)}{\partial \tau }=\frac{\varpi +1\left(\frac{\tau }{\left(\sqrt{\tau^{2}+\xi^{2}}\right)\left(\varpi +1\right)}-\frac{\varpi \tau }{\left(\sqrt{\tau^{2}+\xi^{2}}\right)\varpi^{2}}\right)}{\varpi };\frac{\partial \left(\ln\left(R_{-\frac{1}{2}}\left(\varpi \right)\right)\right)}{\partial \tau }=0.$$

The natural logarithm of likelihood function is differentiated of ξ as the scale parameter:

(A.4.8) $$\begin{array}{c} \frac{\partial \ln L(\theta_{\text{MCPGIGR}})}{\partial \xi }=\sum_{i=1}^{n}\frac{\partial \ln P\left(y_{i}|\beta_{1}^{T},\beta_{2}^{T},\cdots ,\beta_{j}^{T},\tau ,\xi ,\gamma \right)}{\partial \xi }.\\ =\sum_{i=1}^{n}\left(\frac{\partial \left(\sum_{j=2}^{m}\ln K_{{}_{y_{ij}+\gamma }}\left(\sqrt{\varpi \psi^{*}}\right)\right)}{\partial \xi }-\frac{\partial \left(\sum_{j=2}^{m}\left(y_{ij}-y_{i1}\right)\ln\left(R_{\gamma }\left(\varpi \right)\right)\right)}{\partial \xi }-\frac{\partial \left(\sum_{j=2}^{m}\ln K_{\gamma }\left(\varpi \right)\right)}{\partial \xi }+\frac{\partial \left(\sum_{j=2}^{m}\frac{\left(y_{ij}+\gamma \right)}{2}\ln\frac{\varpi }{\psi^{*}}\right)}{\partial \xi }\right)\\ =\sum_{i=1}^{n}(\sum_{j=2}^{m}\left(\frac{\gamma }{\sqrt{\varpi \psi^{*}}}-\frac{K_{{}_{y_{ij}+\gamma +1}}\left(\sqrt{\varpi \psi^{*}}\right)}{K_{{}_{y_{ij}+\gamma }}\left(\sqrt{\varpi \psi^{*}}\right)}\right)\left(\frac{\varpi +\psi^{*}}{2\sqrt{\varpi \psi^{*}}}\right)\left(\frac{\xi }{\sqrt{\tau^{2}+\xi^{2}}}-1\right)-\sum_{j=2}^{m}\left(y_{ij}-y_{i1}\right)\frac{\partial \left(\ln\left(R_{\gamma }\left(\varpi \right)\right)\right)}{\partial \xi }\\ -\left(m-1\right)\left(\frac{\gamma }{\varpi }-\frac{K_{\gamma +1}\left(\varpi \right)}{K_{\gamma }\left(\varpi \right)}\right)\left(\frac{\xi }{\sqrt{\tau^{2}+\xi^{2}}}-1\right)+\sum_{j=2}^{m}\frac{\left(y_{ij}+\gamma \right)}{2}\left(\frac{\xi }{\sqrt{\tau^{2}+\xi^{2}}}-1\right)\left(\frac{1}{\varpi }-\frac{1}{\psi^{*}}\right)) \end{array}$$

with $\varpi =\sqrt{\tau^{2}+\xi^{2}}-\xi$ and $\psi^{*}=2\sum_{j=2}^{m}q_{ij}\exp\left(x_{i}^{T}\beta_{j}\right)+\varpi$, if

$$\begin{array}{c} \frac{\partial \left(\ln\left(R_{-\frac{5}{2}}\left(\varpi \right)\right)\right)}{\partial \xi }=\frac{1}{\varpi^{2}+\varpi }((\frac{2\varpi \left(\frac{\xi }{\sqrt{\tau^{2}+\xi^{2}}}+1\right)+\frac{\xi }{\sqrt{\tau^{2}+\xi^{2}}}+1}{\varpi^{2}+3+3\sqrt{\tau^{2}+\xi^{2}}+3\xi }-\frac{\left(\varpi^{2}+\varpi \right)\left(\left(2\varpi \right)\left(\frac{\xi }{\sqrt{\tau^{2}+\xi^{2}}}+1\right)+\frac{3\xi }{\sqrt{\tau^{2}+\xi^{2}}}+3\right)}{{\left(\varpi^{2}+3+3\sqrt{\tau^{2}+\xi^{2}}+3\xi \right)}^{2}})\\ \times \left(\varpi^{2}+3+3\sqrt{\tau^{2}+\xi^{2}}+3\right));\frac{\partial \left(\ln\left(R_{-\frac{3}{2}}\left(\varpi \right)\right)\right)}{\partial \xi }=\frac{\varpi +1}{\varpi }\left(\frac{\frac{\xi }{\left(\sqrt{\tau^{2}+\xi^{2}}\right)}}{\varpi +1}-\frac{\varpi \left(\frac{\xi }{\sqrt{\tau^{2}+\xi^{2}}}+1\right)}{\varpi^{2}}\right);\frac{\partial \left(\ln\left(R_{-\frac{1}{2}}\left(\varpi \right)\right)\right)}{\partial \xi }=0 \end{array}$$
